# The neurobiology of addiction: the perspective from magnetic resonance imaging present and future

**DOI:** 10.1111/add.13474

**Published:** 2016-07-25

**Authors:** John Suckling, Liam J. Nestor

**Affiliations:** ^1^ Department of Psychiatry and Behavioural and Clinical Neuroscience InstituteUniversity of CambridgeCambridgeUK; ^2^ Cambridge and Peterborough Foundation NHS TrustCambridgeUK; ^3^ Centre for Neuropsychopharmacology, Division of Brain SciencesImperial College LondonLondonUK

**Keywords:** Addiction, arterial spin labelling, blood oxygenation level dependent, brain structure and function, diffussion imaging, functional imaging, magentisation transfer, magnetic resonance imaging, neuroimaging, voxel based morphometry

## Abstract

**Background and Aims:**

Addiction is associated with severe economic and social consequences and personal tragedies, the scientific exploration of which draws upon investigations at the molecular, cellular and systems levels with a wide variety of technologies. Magnetic resonance imaging (MRI) has been key to mapping effects observed at the microscopic and mesoscopic scales. The range of measurements from this apparatus has opened new avenues linking neurobiology to behaviour. This review considers the role of MRI in addiction research, and what future technological improvements might offer.

**Methods:**

A hermeneutic strategy supplemented by an expansive, systematic search of PubMed, Scopus and Web of Science databases, covering from database inception to October 2015, with a conjunction of search terms relevant to addiction and MRI. Formal meta‐analyses were prioritized.

**Results:**

Results from methods that probe brain structure and function suggest frontostriatal circuitry disturbances within specific cognitive domains, some of which predict drug relapse and treatment response. New methods of processing imaging data are opening opportunities for understanding the role of cerebral vasculature, a global view of brain communication and the complex topology of the cortical surface and drug action. Future technological advances include increases in MRI field strength, with concomitant improvements in image quality.

**Conclusions:**

The magnetic resonance imaging literature provides a limited but convergent picture of the neurobiology of addiction as global changes to brain structure and functional disturbances to frontostriatal circuitry, accompanied by changes in anterior white matter.

## Introduction

Without doubt, the technology to image the structure and function of the brain non‐invasively (referred to collectively as neuroimaging) has transformed our understanding of neurological and psychiatric disorders, arguably setting them on a convergent course. Addiction research has benefited greatly from the resurgence of experimental psychology and psychiatry that has been made possible by neuroimaging. Existing reviews of the extant literature have identified some of the major cortical and subcortical components of the complex neurobiological circuitry that forms the substrate for the compulsive seeking, excessive consumption and negative emotions accompanying withdrawal that characterize addictive behaviours. In short, current cumulative knowledge implicates dopaminergic pathways with origins in the ventral tegmental area connected to the prefrontal cortex via the limbic system, in particular the nucleus accumbens, amygdala, ventral pallidum and hippocampus 1, 2, 3, 4, 5.

Measuring and understanding the consequences of the chronic consumption of alcohol by radiological examination has been of interest since at least the 1950s, with generalized atrophy of the brain depicted by pneumoencephalography 6, 7. Modern neuroimaging encompasses a large range of techniques based on a variety of physical phenomena. Although the contribution of radioisotope imaging has been highly significant in the discovery and mapping of the role of dopamine in addiction 8, magnetic resonance imaging (MRI) is the mainstay of addiction imaging research due its safety, absence of radioactivity and flexibility in the information obtained. Advances in MRI technology continue, along with postprocessing algorithms that are creating new representations of MRI data, refreshing the vocabulary describing the brain in health and disorder.

Here, our current knowledge of the neurobiology of addiction is summarized with data from human studies using established methodologies for measuring the structure and function of the brain with MRI. The field is now sufficiently mature that distilling the germane features from the literature can be undertaken by meta‐analysis, and where available these accounts will be drawn upon. With a contemporaneous account of the literature consolidated, recent efforts with new methods and technologies are examined for future opportunities that will increase the breadth of knowledge on the neurobiology of addiction.

## Methods

The objective of this review was a synthesis of the peer‐reviewed literature of the current state of the art of neuroimaging, specifically MRI, in the context of addiction research with a view to projecting future developments in this area.

The primary approach was to assemble the relevant literature with a hermeneutic strategy 9. Here, a small number of seminal papers 1, 2, 3, 4, 5 were identified for review followed by open‐ended searches to locate relevant papers using the citation and author‐tracking functionality of the PubMed (http://www.ncbi.nlm.nih.gov/pubmed), Scopus (http://www.scopus.com) and Web of Science (https://apps.webofknowledge.com/) on‐line databases, and refining the narrative as the content of the evidence became apparent. Additional, expansive explorations were made using a conjunction of the search terms ‘addiction’ or ‘dependency’ and ‘MRI’ or ‘magnetic resonance imaging’ or ‘neuroimaging’, again followed by open‐ended searches of citations and authors. Searching was terminated once a particular line of argument was substantiated. Any formal meta‐analyses that were revealed by these strategies were prioritized in terms of their contribution to the narrative. Key, highly‐cited papers on specific resources, methodologies and techniques acquired from previous qualitative reviews by the authors were also included.

Throughout the search processes no limit was placed on the earliest papers returned to permit a historical perspective. However, the most recent papers included were established by the time of the searches as of October 2015. Only papers with human participants were included. Studies with negative results were incorporated where the complementary, positive results were not supported by overwhelming evidence.

### Current structural and functional MRI findings

#### Brain anatomy and structure

The most prevalent method for measuring brain structure, voxel‐based morphometry (VBM) 10, draws upon the contrast between grey and white matter in high‐resolution MRI to estimate tissue volumes, as well as ventricular volumes that are filled with cerebrospinal fluid (CSF). Following digital alignment of these tissue maps, statistical comparisons that search, for example, for significant case–control differences at every intra‐cerebral location are possible in what is often termed a ‘whole‐brain’ analysis.

Qualitatively, almost all drugs of abuse are associated with reductions in cortical volume, especially in the frontal lobes, reiterating the general observations made in the earliest studies. This is accompanied by increases in the volumes of subcortical structures associated with dependency on stimulants such as cocaine and methamphetamine 11. A formal meta‐analysis of the whole‐brain VBM literature partly supports this view 12 with decreases in volumes in prefrontal cortex, but also in the insula, inferior frontal gyrus, pregenual anterior cingulate and anterior thalamus with, notably, the decline in grey matter in frontal regions related to duration of dependency. However, changes in the subcortices are not seen across the literature, which may be due to inconsistent results in the primary literature or a bias in the sensitivity of the VBM methodology in these regions of the brain 12.

Several studies to date show that different addiction groups have reduced grey matter volume compared to healthy controls in regions of the brain targeted by the specific drug of abuse, including alcohol 13, cocaine 14, 15, amphetamine 16 and nicotine 17, 18, 19. However, the approaches taken in these studies appear to neglect the contribution to structural deficits that arise from relapse, treatment outcome and abstinence; it is unclear how former addicts remain abstinent even with severe structural deficits in brain regions implicated in addiction 20. Furthermore, there is evidence for shared structural deficits between addict and non‐addict populations. Abnormalities in frontostriatal brain systems in both stimulant‐dependent individuals and their biological siblings who have no history of chronic drug abuse 21 potentially devalue the clinical significance of these outcome measures for addiction.

White matter differences have been difficult to observe with whole‐brain VBM in heavy cannabis users 22 and cocaine addiction addicts 23. Detections of white matter changes have been associated with amphetamine use, with results equivocal concerning the direction and location of case–control differences 1. This suggests that if addiction does lead to changes in white matter, they occur most probably at the microscopic scale without consistent observable changes in volume.

#### White matter microstructure

A complementary MRI technique probing brain structure is imaging the diffusion of water. Compared to grey matter, water diffusion has restricted motion in white matter due to the alignment of neuronal fibres. Using this observation, estimates of the trajectories of white matter tracts are possible with a technique known as diffusion tensor imaging (DTI).

DTI produces some of the most spectacular maps of the so‐called human connectome, and has led to changes in our understanding of the way the brain is organized in the healthy population 24. However, there remain some analytical issues that temper confidence in DTI, particularly with estimation of tracts that span large distances 25, 26. An overview of measurable DTI effects in chronic alcoholics draws a picture of reductions in white matter integrity in anterior parts of the brain, particularly the supratentorial and infratentorial white matter bundles, which may be ascribed to myelin degradation 27. However, a more recent study of alcoholics appears to show an opposite pattern of abnormalities 28.

A simplified alternative to reconstructing white matter tracts with DTI is to measure the local direction of water diffusion and the speed of diffusion with the scalar values of fractional anisotropy (FA) and mean diffusivity (MD), respectively. While visualization of white matter connectivity is sacrificed, the established statistical apparatus for testing whole‐brain effects can be deployed in the same way as VBM.

The corpus callosum has come under scrutiny for its role in alcohol addiction from early pathological reports of its reduced volume. A review of DTI data specifically within the corpus callosum 29 points towards decreased FA and increased MD in alcoholics, and a similar reduction in FA in cocaine addicts 23. In both cases, the duration of addictive behaviours appears to amplify these effects, pointing towards a decrease in the density of fibre bundles. These results are in the absence of gross morphological brain changes, indicating that DTI may have enhanced sensitivity relative to VBM.

Reductions in FA in the corpus callosum, as well as the thalamic radiation and inferior longitudinal fasciculus, have also been observed in opiate addicts 30. Again, effects associated with duration of drug use were significant, converging with evidence from an earlier study 31. However, contrary to the regional data, FA in the corpus callosum did not differ between cocaine addicts and control participants when whole‐brain testing for differences. Differences were found in FA in the inferior frontal lobe and increases in the anterior cingulate 23. Other forms of addiction have also been studied with DTI, but without revealing any common underlying pathology. Case–control differences of individuals prone to obsessive gaming on the internet showed increases in FA in the thalamus and posterior regions of the cingulate cortex that do not overlap with the more frontal differences observed with drug addictions 32.

#### Functional response to cognitive tasks

Functional MRI (fMRI) measures brain activity via the blood oxygenation level‐dependent (BOLD) endogenous contrast 33, exploiting the distinct magnetic properties of oxygenated and deoxygenated blood for its detection by MRI. As neuronal activity requires oxygen, the BOLD signal is therefore related indirectly to local functional processing. Experiments with fMRI often induce specific cognitive processes with appropriate stimuli, highlighting the regions and circuits that are involved.

Common to much addictive behaviour is cue–reactivity, in which exposure to stimuli induces craving as a consequence of Pavlovian conditioning 34. A descriptive overview of the literature suggests increased activation in addicted individuals, particularly in prefrontal and orbitofrontal regions 35. However, a formal meta‐analysis of fMRI data 36 reports that regions of increased cue–reactivity converged in nicotine, alcohol and cocaine addicts to the anterior cingulate cortex, amygdala and ventral striatum, which includes the nucleus accumbens. A concurrent meta‐analytical investigation of fMRI and positron emission tomography (PET) studies broadly confirms this pattern 37. Interestingly, several of these areas have also been observed by meta‐analysis as being associated with food cues 38 and, to a lesser extent, in addictive behaviours towards internet gaming 39.

Inhibitory control is the suppression of pre‐potent actions and resisting interference from extraneous stimuli to engage instead in goal‐directed behaviours. In cocaine addicts, increased activity was observed in prefrontal and cingulate cortices, inferior frontal regions and cerebellum during response inhibition, irrespective of whether or not the action was inhibited successfully 40. Conversely, in opiate addicts, activity in anterior cingulate cortex was attenuated during unsuccessful inhibition of response 41.

Functional MRI tasks invoking emotional responses activate the limbic system 42, which is increased in alcoholics when comparing negative to positive valenced images 43, primarily in insula, inferior frontal, medial temporal and parahippocampal regions. A similar task contrast in cocaine addicts also resulted in increased activation, but in parietal and medial prefrontal regions 44.

### Current innovations

Imaging the structure and function of brain has identified key networks associated with addiction. Data processing algorithms are deriving new information from existing data and offering a broader view of the biology of addiction, mechanisms of drug action and potential applications of imaging to clinical management.

#### Brain perfusion

MRI techniques can supplant existing measurements using radioactive tracers, with concomitant improvements in participant safety. One such example is the measurement of blood perfusion with arterial spin labelling (ASL). Arterial blood is ‘labelled’ magnetically as it enters the brain. As the blood perfuses the tissues, the labelling alters the local tissue signal. Subtracting the labelled image from an unlabelled image produces a map of perfusion 45.

Drug dependence results frequently in cardiovascular problems that make up a significant proportion of drug‐related morbidity 46 and effects cognitive performance through cerebral hypoperfusion 47. Heart disease is well‐known in chronic alcoholism 48, and the perfusion of frontal and parietal grey matter measured in case–control studies by ASL in alcoholics is significantly lower 49. Similarly, in opiate users administration of heroin reduces perfusion in the insula, anterior cingulate and medial prefrontal cortices 50, and in a separate study more severe depressive symptoms are associated with reduced perfusion in prefrontal and middle frontal regions 51. Internet gaming addiction demonstrates a more mixed picture, with both increases and decreases in perfusion widespread throughout the neocortex 52, suggesting a quite different disorder from that engendered by substance dependency.

#### The functionally connected brain

It has become known recently that brain networks not only appear in response to stimuli but are, in fact, configured and poised continually to receive input 53. This remarkable transformation in the way the brain is conceived has had a profound effect on experimental design, with interest now on the so‐called ‘resting‐state’ or, more precisely, data acquisition in the absence of any specific external stimulus.

Functional connectivity between two or more brain regions refers frequently to the Pearson's correlation coefficient between corresponding BOLD time–series. A hypothesis‐driven approach is to select a particular region, known as the ‘seed’, and then calculate the correlation between this region and every other location within a brain network, testing subsequently for statistical differences.

Prevailing ideas on the neurocircuitry of addiction have centred on dopaminergic pathways in the mesocorticolimbic pathway, with projections to the prefrontal cortex. This is an inviting target for seed‐based connectivity analysis, and the literature has become rapidly populated with studies of this type (reviewed in 54). In general, alterations to this circuit are found consistently across addictions to different drugs, although the direction of this effect is at times inconsistent, particularly between striatal regions and the prefrontal cortex.

If functional connections between all regions are calculated they can be represented as a network constructed from nodes (brain regions) linked by edges (significant functional connectivity). Topological properties of the network include measures of the global efficiency of communication, local concentration of connections, overall organizational structure, and so on 55. Application of this approach to addiction research is under‐represented to date, although preliminary data indicate some sensitivity.

In opiate users, networks had increased connectivity between neighbouring brain regions with an increased number of connections in regions overlapping with the mesocorticolimbic pathway; namely, components of the striatum and prefrontal cortex. Frontal and cingulate cortices, thalamus, cerebellum and areas of the temporal and parietal lobes also had increased connectivity implicating networks that mediate motivation, memory and learning 56. By way of contrast, a similar analysis on internet addiction did not discover any overall change in topology. Nevertheless, localized increases in connectivity were routed through the parietal lobe, limbic system (middle and anterior cingulate cortex) and thalamus 57, suggesting some overlap in the substrate of addiction measured by the connectome.

#### Surface models of the cortex

A mesh representation of the grey matter mantle covering the cerebral hemispheres can be estimated from the inner (grey–white boundary) and outer (pial) surfaces. Measures of cortical thickness, volume and geometry can then be made that are complementary to volume estimates from VBM, which is a combination of thickness, surface area and gyrification 58.

Thinner cortices in cocaine addicts are seen in dorsal prefrontal and insula cortices, elements of the brain reward circuit that is also implicated by VBM, and are accompanied by an overall reduction in cortical volume 59. However, amphetamine use is not related directly to a reduction in cortical thickness, but with comorbid alcohol consumption when there is thinning in frontal and pre‐central regions 60. Alcoholism in adults is itself linked to decreases in cortical thickness in frontal, pre‐ and postcentral, temporal and occipital cortical regions and correlated positively with severity of abuse 61. Reduced cingulate cortex and inferior frontal gyrus thickness in adolescents appear to be predictive markers for transition to addictive behaviour, when subcortical and temporal regions are reduced further 62. Interestingly, the lateral orbitofrontal cortex is also reduced in adolescents with internet addiction 63, indicating some similarities with a predisposition to alcohol abuse.

#### Pharmacological MRI

Pharmacological MRI (phMRI) is used to test how pharmacological agents modulate brain activation and/or cognitive performance during an fMRI study. Drugs are usually given in an acute challenge (i.e. as a single dose prior to the scanning session), although recently chronic drug treatment regimens have been used to mimic more closely the clinical outcome of drug administration 64. Importantly, the use of phMRI may have some value with respect to neuroimaging biomarkers for addiction 65. Here, such challenge studies may be able to demonstrate that patterns of activation in early drug abstinence are able to predict whether some addicts are likely to respond to a particular class of psychotrophic, should it be developed as a medication for addiction and relapse prevention. The use of phMRI may allow neuroscience research in the addictions to make more informed inferences regarding the neurochemistry of underlying disturbances to particular cognitive processes in addiction 66, 67, 68.

#### Functional biomarkers for treatment monitoring

Addiction is associated commonly with increased brain activity to drug‐related cues 69, 70, 71, 72, 73, 74, 75 and reduced activity to cues that predict non‐drug rewards 76, 77, 78, 79, 80, 81, together with reduced activity in response to the demands of executive control 41, 82, 83, 84, 85, 86 and social cognition 87. Examining neural responses within these domains is of particular interest with respect to the development of fMRI biomarkers that identify neural mechanisms for drug relapse during early abstinence, and for the treatment of addiction. There may be a common fMRI ‘signature’ across cognitive domains within loci that may be explored as potential targets for cognitive 88, 89, 90 and/or pharmacological 71, 90, 91, 92, 93 therapies in the addictions. This would be especially significant if distinct neural brain biomarkers could predict both successful and unsuccessful abstinence and treatment response.

Studies that have used fMRI to predict relapse and treatment response have been surprisingly scarce, but endorse imaging in addiction research. A seminal study of this type was conducted in methamphetamine addicts during a decision‐making task. Activation patterns in the insular, posterior cingulate and temporal cortex obtained in early recovery predicted correctly 90% of participants who did not relapse and 94% of participants who did 94. In a longitudinal study examining neural responses during reinforcement learning as a predictor of relapse in methamphetamine addiction, individuals who were abstinent and then relapsed 1 year later showed greater prefrontal activation during learning, but attenuated striatal, insular and frontal activation in response to feedback compared with those methamphetamine‐dependent people who remained abstinent 95. In a study exploiting the processing of non‐drug‐related stimuli, higher activation in the striatum and insula in response to potentially risky rewards during in‐patient treatment was found only in those patients who subsequently remained drug abstinent 96. There are also several other studies which have demonstrated how BOLD activation patterns are able to predict relapse in addiction populations 97, 98, 99, 100.

FMRI has also been used to predict treatment responses to medications in addiction. In alcoholic patients, the treatment response to naltrexone has been shown to be predicted by the level of pre‐treatment cue–reactivity in the ventral striatum in response to alcohol‐related cues 101. Therefore, while somewhat scarce, there is good scientific evidence that endorses the use of fMRI for the identification of potential neural biomarkers for abstinence, relapse and treatment outcome in addiction. The search for such fMRI biomarkers is highly desirable as a prelude to the development of new interventions in addiction.

### Future directions

There have been large improvements in MRI technology as a result of the evolution of precision engineering and electronics, with a trend towards increases in the static magnetic field of scanners to 7 T or greater, and the simultaneous acquisition of MRI data with physiological information available from radioligand imaging. Optimization of existing techniques will certainly yield the expected improvements in spatial resolution, signal‐to‐noise and increased sensitivity 102, as well as the development of new protocols to detect drug actions.

#### Quantitative structural relaxometry

In general, MRI scans depicting brain structure are not associated with SI units and are subject to variations in scanner hardware and software, environmental changes in the scanning room and so on, over and above the physiological variations that may be of interest. This is problematic for longitudinal MRI assessments and when combining data in multi‐centre studies 103.

Designed for the measurement of absolute values of relaxation rates, MRI relaxometry makes several rapid acquisitions systematically varying key parameters. Variants of the technique have been shown to have excellent characteristics in comparisons across multiple scanning centres 104, 105, with benefits to all areas of clinical neuroscience research. Of particular relevance to addiction, MRI relaxometry also makes possible quantitative measurement of myelin concentration through a process known as magnetization transfer. Protons in water molecules have their magnetic resonance properties altered by being bound to macromolecules such as myelin, assumed to be the predominant compound of this type in the brain, in a way that is related to its concentration 106. We have already seen from diffusion imaging that white matter degradation is significant in addiction, and that myelin loss may be part of the underlying process. Magnetization transfer, and MRI relaxometry in general, provide the means to follow the trajectory of brain changes reliably during drug‐taking, remission and relapse as a potential predictor of their onset.

#### Susceptibility weighted venography

Magnetic susceptibility describes the behaviour of a material when placed in a magnetic field. A good example of this is shown in both deoxygenated and oxygenated haemoglobin, which have contrasting magnetic properties, and is the physical origin of the BOLD effect. It is also an effect that is exploited to obtain contrast between deoxygenated blood‐filled vessels and the surrounding tissue, and thus visualize the venal system in fine detail 107.

Cardiovascular illness is a major comoribity of addiction 46, and changes in cerebral blood flow are well known as a consequence of drug use 108, which may lead to an increased risk of intra‐cerebral haemorrhage 109 detectable by susceptibility imaging. At increased MRI field strengths, susceptibility imaging is capable of detecting vessels < 1 mm in small structures relevant to addiction research, such as the thalamus 110 and midbrain 111, with possibilities for a more holistic understanding of the neurobiology of addiction.

#### Simultaneous MRI and PET

Most fMRI studies do not consider the problem of reverse inference; in other words, specific functional significance ascribed to fMRI activations on the basis of the known distribution of neurotransmitters and receptors. One common example of this concerns reports describing reductions in the BOLD signal in dopamine‐rich areas of the brain (e.g. nucleus accumbens) in response to a non‐drug reward (e.g. money) in an addiction population that is then attributed to deficits in this particular neurotransmitter. In fact, fMRI cannot make direct inferences about the neurochemistry underlying a particular functional process and how disturbances associated with addiction are alterations of this neurochemistry.

The advent of combined MRI and PET imaging systems can potentially overcome this problem. Although very much in its infancy, PET–MRI has the potential to demonstrate specifically that reductions in the BOLD signal in response to a non‐drug reward are in brain regions that are the result of simultaneous and colocated reductions in dopamine release. Initial PET–MRI studies have already demonstrated that these complementary measures of brain function can provide new insights into the functional and structural organization of the brain 112. This methodology is also likely to revolutionize drug development strategies in addiction and potentially reduce the high costs incurred in drug discovery programmes.

## Concluding remarks

These are pioneering days for the investigation of addiction with neuroimaging. Individual reports across a wide range of measures of brain structure and function are often difficult to reconcile, or even contradictory. However, where the literature is reasonably well populated a more convergent picture is developing that encourages continued effort with the promise of a coherent narrative for this disorder. Imaging studies paint a picture of addiction as global changes to brain structure and functional disturbances to frontostriatal circuitry, accompanied by changes in anterior white matter. However, it is unlikely that neuroimaging alone will provide the evidence for a unifying theory of addiction. The wide variety of drugs or activities that are included within the description of addiction, and the physical and mental comorbidities that cause or are caused by addictive behaviours, contribute to the observed heterogeneity, emphasizing the importance of sample selection and participant stratification.

It might be argued that before rushing to meet the future, addiction research might be better served by consolidating what is already known and available. Meta‐analyses have proved to be a vital tool in other areas of neuroimaging research, consolidating the evidence and streamlining the prevailing narrative 113. Development of meta‐analytical algorithms offer improved sensitivity, but obviously can only be as convincing as the quality and quantity of the primary literature permit. Increasing confidence in the literature on addiction could be facilitated by larger sample sizes in multi‐centre studies, but also by open access to appropriate imaging data repositories, following the successes of initiatives for conditions such as autism 114 and Alzheimer's disease 115.

Clearly, MRI imaging is only one part of a wider effort to understand the aetiology and treatment of addiction. For brevity this review has not made reference to the literature using magnetic resonance spectroscopy 11 or, indeed, other imaging modalities. This is not to downplay their importance. What unites all these techniques, at least in the short to medium term, is that the greatest advances may not come from new measurements, but from applying innovations in data processing and computer modelling. Nevertheless, new techniques will lead to new opportunities. The monitoring and prediction of abstinence and relapse are key to supporting clinical decision‐making, and the discovery of biomarkers that are sensitive to pharmacological or psychological therapies would be a significant step forward.

## Declaration of interests

J.S. has received research funds from GlaxoSmithKline for data analysis of experimental medicine studies and clinical trials.

## References

[1] Berman S. , O'Neill J. , Fears S. , Bartzokis G. , London E. D. Abuse of amphetamines and structural abnormalities in the brain. Addict Rev 2008; 1141: 95–220. 10.1196/annals.1441.031 PMC2769923 18991959

[2] Duncan J. R. Current perspectives on the neurobiology of drug addiction: a focus on genetics and factors regulating gene expression. ISRN Neurol 2012; 2012: 972607. 23097719 10.5402/2012/972607 PMC3477671

[3] Goldstein R. Z. , Volkow N. D. Dysfunction of the prefrontal cortex in addiction: neuroimaging findings and clinical implications. Nat Rev Neurosci 2011; 12: 652–69. 22011681 10.1038/nrn3119 PMC3462342

[4] Lingford‐Hughes A. , Nutt D. Neurobiology of addiction and implications for treatment. Br J Psychiatry 2003; 182: 97–100. 12562734 10.1192/bjp.182.2.97

[5] Koob G. F. , Simon E. J. The neurobiology of addiction: where we have been and where we are going. J Drug Issues 2009; 39: 115–32. 20622969 10.1177/002204260903900110 PMC2901107

[6] Kirsher J. P. , Pierson C. A. Les atrophies cerebrales dans les toxicomanies. Role de la pneumoencephalographie. [Cerebral atrophy in the addictions. The role of pneumoencephalography] Maroc Med 1956; 35: 668. 13368675

[7] Pierson C. A. , Kirsher J. P. La pneumoencephalographie chez l'alcoolinque. Rev Neurol 1954; 90: 673. 13225272

[8] Volkow N. D. , Fowler J. S. , Wang G. J. , Swanson J. M. , Telang F. Dopamine in drug abuse and addiction: results of imaging studies and treatment implications. Arch Neurol 2007; 64: 1575–9. 17998440 10.1001/archneur.64.11.1575

[9] Boell S. K. , Cecez‐Kecmanovic D. Literature reviews and the hermeneutic circle. Aust Acad Res Libraries 2010; 41: 129–44.

[10] Mechelli A. , Price C. J. , Friston K. J. , Ashburner J. Voxel‐based morphometry of the human brain: methods and applications. Curr Med Imag Rev 2005; 1: 105–13.

[11] Fowler J. S. , Volkow N. D. , Kassed C. A. , Chang L. Imaging the addicted human brain. Sci Pract Perspect 2007; 3: 4–16. 17514067 10.1151/spp07324 PMC2851068

[12] Ersche K. D. , Williams G. B. , Robbins T. W. , Bullmore E. T. Meta‐analysis of structural brain abnormalities associated with stimulant drug dependence and neuroimaging of addiction vulnerability and resilience. Curr Opin Neurobiol 2013; 23: 615–24. 23523373 10.1016/j.conb.2013.02.017

[13] Asensio S. , Morales J. L. , Senabre I. , Romero M. J. , Beltran M. A. , Flores‐Bellver M. *et al.* Magnetic resonance imaging structural alterations in brain of alcohol abusers and its association with impulsivity. Addict Biol 2016; 21: 962–71. 25988724 10.1111/adb.12257

[14] Connolly C. G. , Bell R. P. , Foxe J. J. , Garavan H. Dissociated grey matter changes with prolonged addiction and extended abstinence in cocaine users. PLOS ONE 2013; 8: e59645. 10.1371/journal.pone.0059645 PMC3601087 23527239

[15] Tanabe J. , Tregellas J. R. , Dalwani M. , Thompson L. , Owens E. , Crowley T. *et al.* Medial orbitofrontal cortex gray matter is reduced in abstinent substance‐dependent individuals. Biol Psychiatry 2009; 65: 160–4. 18801475 10.1016/j.biopsych.2008.07.030 PMC2640220

[16] Daumann J. , Koester P. , Becker B. , Wagner D. , Imperati D. , Gouzoulis‐Mayfrank E. *et al.* Medial prefrontal gray matter volume reductions in users of amphetamine‐type stimulants revealed by combined tract‐based spatial statistics and voxel‐based morphometry. Neuroimage 2011; 54: 794–801. 20817105 10.1016/j.neuroimage.2010.08.065

[17] Stoeckel L. E. , Chai X. J. , Zhang J. , Whitfield‐Gabrieli S. , Evins A. E. Lower gray matter density and functional connectivity in the anterior insula in smokers compared with never smokers. Addict Biol 2016; 21: 972–81. 25990865 10.1111/adb.12262 PMC4654721

[18] Gallinat J. , Meisenzahl E. , Jacobsen L. K. , Kalus P. , Bierbrauer J. , Kienast T. *et al.* Smoking and structural brain deficits: a volumetric MR investigation. Eur J Neurosci 2006; 24: 1744–50. 17004938 10.1111/j.1460-9568.2006.05050.x

[19] Brody A. L. , Mandelkern M. A. , Jarvik M. E. , Lee G. S. , Smith E. C. , Huang J. C. *et al.* Differences between smokers and nonsmokers in regional gray matter volumes and densities. Biol Psychiatry 2004; 55: 77–84. 14706428 10.1016/s0006-3223(03)00610-3

[20] Wang L. , Zou F. , Zhai T. , Lei Y. , Tan S. , Jin X. *et al.* Abnormal gray matter volume and resting‐state functional connectivity in former heroin‐dependent individuals abstinent for multiple years. Addict Biol 2016; 21: 646–56. 25727574 10.1111/adb.12228

[21] Ersche K. D. , Jones P. S. , Williams G. B. , Turton A. J. , Robbins T. W. , Bullmore E. T. Abnormal brain structure implicated in stimulant drug addiction. Science 2012; 335: 601–4. 22301321 10.1126/science.1214463

[22] Cousijn J. , Wiers R. W. , Ridderinkhof K. R. , van den Brink W. , Veltman D. J. , Goudriaan A. E. Grey matter alterations associated with cannabis use: results of a VBM study in heavy cannabis users and healthy controls. Neuroimage 2012; 59: 3845–51. 21982932 10.1016/j.neuroimage.2011.09.046

[23] Romero M. J. , Asensio S. , Palau C. , Sanchez A. , Romero F. J. Cocaine addiction: diffusion tensor imaging study of the inferior frontal and anterior cingulate white matter. Psychiatry Res 2010; 181: 57–63. 19959341 10.1016/j.pscychresns.2009.07.004

[24] van den Heuvel M. P. , Sporns O. Rich‐club organization of the human connectome. J Neurosci 2011; 31: 15775–86. 22049421 10.1523/JNEUROSCI.3539-11.2011 PMC6623027

[25] Deco G. , McIntosh A. R. , Shen K. , Hutchison R. M. , Menon R. S. , Everling S. *et al.* Identification of optimal structural connectivity using functional connectivity and neural modeling. J Neurosci 2014; 34: 7910–6. 24899713 10.1523/JNEUROSCI.4423-13.2014 PMC6608269

[26] Vaessen M. J. , Hofman P. A. , Tijssen H. N. , Aldenkamp A. P. , Jansen J. F. , Backes W. H. The effect and reproducibility of different clinical DTI gradient sets on small world brain connectivity measures. Neuroimage 2010; 51: 1106–16. 20226864 10.1016/j.neuroimage.2010.03.011

[27] Chanraud S. , Zahr N. , Sullivan E. V. , Pfefferbaum A. MR diffusion tensor imaging: a window into white matter integrity of the working brain. Neuropsychol Rev 2010; 20: 209–25. 20422451 10.1007/s11065-010-9129-7 PMC2910550

[28] Schulte T. , Muller‐Oehring E. M. , Pfefferbaum A. , Sullivan E. V. Neurocircuitry of emotion and cognition in alcoholism: contributions from white matter fiber tractography. Dialogues Clin Neurosci 2010; 12: 554–60. 21319499 10.31887/DCNS.2010.12.4/tschulte PMC3181985

[29] Arnone D. , Abou‐Saleh M. T. , Barrick T. R. Diffusion tensor imaging of the corpus callosum in addiction. Neuropsychobiology 2006; 54: 107–13. 17108711 10.1159/000096992

[30] Bora E. , Yucel M. , Fornito A. , Pantelis C. , Harrison B. J. , Cocchi L. *et al.* White matter microstructure in opiate addiction. Addict Biol 2012; 17: 141–8. 21070508 10.1111/j.1369-1600.2010.00266.x

[31] Liu P. , Lu H. , Filbey F. M. , Tamminga C. A. , Cao Y. , Adinoff B. MRI assessment of cerebral oxygen metabolism in cocaine‐addicted individuals: hypoactivity and dose dependence. NMR Biomed 2014; 27: 726–32. 24757009 10.1002/nbm.3114 PMC4084967

[32] Dong G. , DeVito E. , Huang J. , Du X. Diffusion tensor imaging reveals thalamus and posterior cingulate cortex abnormalities in internet gaming addicts. J Psychiatr Res 2012; 46: 1212–6. 22727905 10.1016/j.jpsychires.2012.05.015 PMC3650484

[33] Ogawa S. , Menon R. S. , Tank D. W. , Kim S. G. , Merkle H. , Ellermann J. M. *et al.* Functional brain mapping by blood oxygenation level‐dependent contrast magnetic‐resonance‐imaging—a comparison of signal characteristics with a biophysical model. Biophys J 1993; 64: 803–12. 8386018 10.1016/S0006-3495(93)81441-3 PMC1262394

[34] Carter B. L. , Tiffany S. T. Meta‐analysis of cue‐reactivity in addiction research. Addiction 1999; 94: 327–40. 10605857

[35] Wilson S. J. , Sayette M. A. , Fiez J. A. Prefrontal responses to drug cues: a neurocognitive analysis. Nat Neurosci 2004; 7: 211–4. 15001989 10.1038/nn1200 PMC2637355

[36] Kuhn S. , Gallinat J. Common biology of craving across legal and illegal drugs ‐ a quantitative meta‐analysis of cue–reactivity brain response. Eur J Neurosci 2011; 33: 1318–26. 21261758 10.1111/j.1460-9568.2010.07590.x

[37] Chase H. W. , Eickhoff S. B. , Laird A. R. , Hogarth L. The neural basis of drug stimulus processing and craving: an activation likelihood estimation meta‐analysis. Biol Psychiatry 2011; 70: 785–93. 21757184 10.1016/j.biopsych.2011.05.025 PMC4827617

[38] Tang D. W. , Fellows L. K. , Small D. M. , Dagher A. Food and drug cues activate similar brain regions: a meta‐analysis of functional MRI studies. Physiol Behav 2012; 106: 317–24. 22450260 10.1016/j.physbeh.2012.03.009

[39] Meng Y. , Deng W. , Wang H. , Guo W. , Li T. The prefrontal dysfunction in individuals with internet gaming disorder: a meta‐analysis of functional magnetic resonance imaging studies. Addict Biol 2014; 20: 799–808. 24889021 10.1111/adb.12154

[40] Connolly C. G. , Foxe J. J. , Nierenberg J. , Shpaner M. , Garavan H. The neurobiology of cognitive control in successful cocaine abstinence. Drug Alcohol Depend 2012; 121: 45–53. 21885214 10.1016/j.drugalcdep.2011.08.007 PMC3262906

[41] Forman S. D. , Dougherty G. G. , Casey B. J. , Siegle G. J. , Braver T. S. , Barch D. M. *et al.* Opiate addicts lack error‐dependent activation of rostral anterior cingulate. Biol Psychiatry 2004; 55: 531–7. 15023582 10.1016/j.biopsych.2003.09.011

[42] Phan K. L. , Wager T. , Taylor S. F. , Liberzon I. Functional neuroanatomy of emotion: a meta‐analysis of emotion activation studies in PET and fMRI. Neuroimage 2002; 16: 331–48. 12030820 10.1006/nimg.2002.1087

[43] Gilman J. M. , Hommer D. W. Modulation of brain response to emotional images by alcohol cues in alcohol‐dependent patients. Addict Biol 2008; 13: 423–34. 18507736 10.1111/j.1369-1600.2008.00111.x

[44] Asensio S. , Romero M. J. , Palau C. , Sanchez A. , Senabre I. , Morales J. L. *et al.* Altered neural response of the appetitive emotional system in cocaine addiction: an fMRI Study. Addict Biol 2010; 15: 504–16. 20579005 10.1111/j.1369-1600.2010.00230.x

[45] van Laar P. J. , van der Grond J. , Hendrikse J. Brain perfusion territory imaging: methods and clinical applications of selective arterial spin‐labeling MR imaging. Radiology 2008; 246: 354–64. 18227536 10.1148/radiol.2462061775

[46] Ghuran A. , Nolan J. The cardiac complications of recreational drug use. West J Med 2000; 173: 412–5. 11112762 10.1136/ewjm.173.6.412 PMC1071198

[47] Alosco M. L. , Gunstad J. , Jerskey B. A. , Xu X. M. , Clark U. S. , Hassenstab J. *et al.* The adverse effects of reduced cerebral perfusion on cognition and brain structure in older adults with cardiovascular disease. Brain Behav 2013; 3: 626–36. 24363966 10.1002/brb3.171 PMC3868168

[48] Knochel J. P. Cardiovascular effects of alcohol. Ann Intern Med 1983; 98: 849–54. 6847024 10.7326/0003-4819-98-5-849

[49] Mon A. , Durazzo T. C. , Gazdzinski S. , Meyerhoff D. J. The impact of chronic cigarette smoking on recovery from cortical gray matter perfusion deficits in alcohol dependence: longitudinal arterial spin labeling MRI. Alcohol Clin Exp Res 2009; 33: 1314–21. 19413652 10.1111/j.1530-0277.2009.00960.x PMC2975051

[50] Denier N. , Gerber H. , Vogel M. , Klarhofer M. , Riecher‐Rossler A. , Wiesbeck G. A. *et al.* Reduction in cerebral perfusion after heroin administration: a resting state arterial spin labeling Study. PLOS ONE 2013; 8: e71461. 10.1371/journal.pone.0071461 PMC3769358 24039715

[51] Suh J. J. , Langleben D. D. , Ehrman R. N. , Hakun J. G. , Wang Z. , Li Y. *et al.* Low prefrontal perfusion linked to depression symptoms in methadone‐maintained opiate‐dependent patients. Drug Alcohol Depend 2009; 99: 11–7. 18674871 10.1016/j.drugalcdep.2008.06.007 PMC2673981

[52] Feng Q. , Chen X. , Sun J. , Zhou Y. , Sun Y. , Ding W. *et al.* Voxel‐level comparison of arterial spin‐labeled perfusion magnetic resonance imaging in adolescents with internet gaming addiction. Behav Brain Funct 2013; 9: 33. 23937918 10.1186/1744-9081-9-33 PMC3751515

[53] Cole M. W. , Bassett D. S. , Power J. D. , Braver T. S. , Petersen S. E. Intrinsic and task‐evoked network architectures of the human brain. Neuron 2014; 83: 238–51. 24991964 10.1016/j.neuron.2014.05.014 PMC4082806

[54] Sutherland M. T. , McHugh M. J. , Pariyadath V. , Stein E. A. Resting state functional connectivity in addiction: lessons learned and a road ahead. Neuroimage 2012; 62: 2281–95. 22326834 10.1016/j.neuroimage.2012.01.117 PMC3401637

[55] Bullmore E. , Sporns O. Complex brain networks: graph theoretical analysis of structural and functional systems. Nat Rev Neurosci 2009; 10: 186–98. 19190637 10.1038/nrn2575

[56] Yuan K. , Qin W. , Liu J. , Guo Q. , Dong M. , Sun J. *et al.* Altered small‐world brain functional networks and duration of heroin use in male abstinent heroin‐dependent individuals. Neurosci Lett 2010; 477: 37–42. 20417253 10.1016/j.neulet.2010.04.032

[57] Wee C. Y. , Zhao Z. , Yap P. T. , Wu G. , Shi F. , Price T. *et al.* Disrupted brain functional network in internet addiction disorder: a resting‐state functional magnetic resonance imaging study. PLOS ONE 2014; 9: e107306. 10.1371/journal.pone.0107306 PMC4165900 25226035

[58] Hutton C. , Draganski B. , Ashburner J. , Weiskopf N. A comparison between voxel‐based cortical thickness and voxel‐based morphometry in normal aging. Neuroimage 2009; 48: 371–80. 19559801 10.1016/j.neuroimage.2009.06.043 PMC2741580

[59] Makris N. , Gasic G. P. , Kennedy D. N. , Hodge S. M. , Kaiser J. R. , Lee M. J. *et al.* Cortical thickness abnormalities in cocaine addiction—a reflection of both drug use and a pre‐existing disposition to drug abuse? Neuron 2008; 60: 174–88. 18940597 10.1016/j.neuron.2008.08.011 PMC3772717

[60] Lawyer G. , Bjerkan P. S. , Hammarberg A. , Jayaram‐Lindstrom N. , Franck J. , Agartz I. Amphetamine dependence and co‐morbid alcohol abuse: associations to brain cortical thickness. BMC Pharmacol 2010; 10: 5. 20487539 10.1186/1471-2210-10-5 PMC2883539

[61] Fortier C. B. , Leritz E. C. , Salat D. H. , Venne J. R. , Maksimovskiy A. L. , Williams V. *et al.* Reduced cortical thickness in abstinent alcoholics and association with alcoholic behavior. Alcohol Clin Exp Res 2011; 35: 2193–201. 21919920 10.1111/j.1530-0277.2011.01576.x PMC3613762

[62] Squeglia L. M. , Rinker D. A. , Bartsch H. , Castro N. , Chung Y. , Dale A. M. *et al.* Brain volume reductions in adolescent heavy drinkers. Dev Cogn Neurosci 2014; 9: 117–25. 24632141 10.1016/j.dcn.2014.02.005 PMC4061267

[63] Hong S. B. , Kim J. W. , Choi E. J. , Kim H. H. , Suh J. E. , Kim C. D. *et al.* Reduced orbitofrontal cortical thickness in male adolescents with internet addiction. Behav Brain Funct 2013; 9: 11. 23497383 10.1186/1744-9081-9-11 PMC3608995

[64] Lukas S. E. , Lowen S. B. , Lindsey K. P. , Conn N. , Tartarini W. , Rodolico J. *et al.* Extended‐release naltrexone (XR‐NTX) attenuates brain responses to alcohol cues in alcohol‐dependent volunteers: a bold FMRI study. Neuroimage 2013; 78: 176–85. 23571420 10.1016/j.neuroimage.2013.03.055

[65] Paterson L. M. , Flechais R. S. , Murphy A. , Reed L. J. , Abbott S. , Boyapati V. *et al.* The Imperial College Cambridge Manchester (ICCAM) platform study: an experimental medicine platform for evaluating new drugs for relapse prevention in addiction. Part A: Study description. J Psychopharmacol 2015; 29: 943–60. 10.1177/0269881115596155 26246443

[66] Ersche K. D. , Roiser J. P. , Abbott S. , Craig K. J. , Muller U. , Suckling J. *et al.* Response perseveration in stimulant dependence is associated with striatal dysfunction and can be ameliorated by a D‐2/3 receptor agonist. Biol Psychiatry 2011; 70: 754–62. 21967987 10.1016/j.biopsych.2011.06.033

[67] Schmaal L. , Joos L. , Koeleman M. , Veltman D. J. , van den Brink W. , Goudriaan A. E. Effects of modafinil on neural correlates of response inhibition in alcohol‐dependent patients. Biol Psychiatry 2013; 73: 211–8. 22858150 10.1016/j.biopsych.2012.06.032

[68] Ghahremani D. G. , Tabibnia G. , Monterosso J. , Hellemann G. , Poldrack R. A. , London E. D. Effect of modafinil on learning and task‐related brain activity in methamphetamine‐dependent and healthy individuals. Neuropsychopharmacology 2011; 36: 950–9. 21289606 10.1038/npp.2010.233 PMC3077264

[69] David S. P. , Munafo M. R. , Johansen‐Berg H. , Smith S. M. , Rogers R. D. , Matthews P. M. *et al.* Ventral striatum/nucleus accumbens activation to smoking‐related pictorial cues in smokers and nonsmokers: a functional magnetic resonance imaging study. Biol Psychiatry 2005; 58: 488–94. 16023086 10.1016/j.biopsych.2005.04.028 PMC4439461

[70] Due D. L. , Huettel S. A. , Hall W. G. , Rubin D. C. Activation in mesolimbic and visuospatial neural circuits elicited by smoking cues: evidence from functional magnetic resonance imaging. Am J Psychiatry 2002; 159: 954–60. 12042183 10.1176/appi.ajp.159.6.954

[71] Franklin T. , Wang Z. , Suh J. J. , Hazan R. , Cruz J. , Li Y. *et al.* Effects of varenicline on smoking cue‐triggered neural and craving responses. Arch Gen Psychiatry 2011; 68: 516–26. 21199958 10.1001/archgenpsychiatry.2010.190 PMC3743240

[72] Franklin T. R. , Wang Z. , Wang J. , Sciortino N. , Harper D. , Li Y. *et al.* Limbic activation to cigarette smoking cues independent of nicotine withdrawal: a perfusion fMRI study. Neuropsychopharmacology 2007; 32: 2301–9. 17375140 10.1038/sj.npp.1301371

[73] Garavan H. , Pankiewicz J. , Bloom A. , Cho J. K. , Sperry L. , Ross T. J. *et al.* Cue‐induced cocaine craving: neuroanatomical specificity for drug users and drug stimuli. Am J Psychiatry 2000; 157: 1789–98. 11058476 10.1176/appi.ajp.157.11.1789

[74] Goldstein R. Z. , Tomasi D. , Rajaram S. , Cottone L. A. , Zhang L. , Maloney T. *et al.* Role of the anterior cingulate and medial orbitofrontal cortex in processing drug cues in cocaine addiction. Neuroscience 2007; 144: 1153–9. 17197102 10.1016/j.neuroscience.2006.11.024 PMC1852512

[75] Janes A. C. , Pizzagalli D. A. , Richardt S. , Frederick Bde B. , Holmes A. J. , Sousa J. *et al.* Neural substrates of attentional bias for smoking‐related cues: an FMRI study. Neuropsychopharmacology 2010; 35: 2339–45. 20703221 10.1038/npp.2010.103 PMC2955848

[76] Buhler M. , Vollstadt‐Klein S. , Kobiella A. , Budde H. , Reed L. J. , Braus D. F. *et al.* Nicotine dependence is characterized by disordered reward processing in a network driving motivation. Biol Psychiatry 2010; 67: 745–52. 20044075 10.1016/j.biopsych.2009.10.029

[77] Diekhof E. K. , Falkai P. , Gruber O. Functional neuroimaging of reward processing and decision‐making: a review of aberrant motivational and affective processing in addiction and mood disorders. Brain Res Rev 2008; 59: 164–84. 18675846 10.1016/j.brainresrev.2008.07.004

[78] Peters J. , Bromberg U. , Schneider S. , Brassen S. , Menz M. , Banaschewski T. *et al.* Lower ventral striatal activation during reward anticipation in adolescent smokers. Am J Psychiatry 2011; 168: 540–9. 21362742 10.1176/appi.ajp.2010.10071024

[79] Wrase J. , Schlagenhauf F. , Kienast T. , Wustenberg T. , Bermpohl F. , Kahnt T. *et al.* Dysfunction of reward processing correlates with alcohol craving in detoxified alcoholics. Neuroimage 2007; 35: 787–94. 17291784 10.1016/j.neuroimage.2006.11.043

[80] Gradin V. B. , Baldacchino A. , Balfour D. , Matthews K. , Steele J. D. Abnormal brain activity during a reward and loss task in opiate‐dependent patients receiving methadone maintenance therapy. Neuropsychopharmacology 2014; 39: 885–94. 24132052 10.1038/npp.2013.289 PMC3924523

[81] Bustamante J. C. , Barros‐Loscertales A. , Costumero V. , Fuentes‐Claramonte P. , Rosell‐Negre P. , Ventura‐Campos N. *et al.* Abstinence duration modulates striatal functioning during monetary reward processing in cocaine patients. Addict Biol 2014; 19: 885–94. 23445167 10.1111/adb.12041

[82] Ersche K. D. , Fletcher P. C. , Lewis S. J. , Clark L. , Stocks‐Gee G. , London M. *et al.* Abnormal frontal activations related to decision‐making in current and former amphetamine and opiate dependent individuals. Psychopharmacology (Berl) 2005; 180: 612–23. 16163533 10.1007/s00213-005-2205-7 PMC3664787

[83] Gowin J. L. , Mackey S. , Paulus M. P. Altered risk‐related processing in substance users: imbalance of pain and gain. Drug Alcohol Depend 2013; 132: 13–21. 23623507 10.1016/j.drugalcdep.2013.03.019 PMC3748224

[84] Fishbein D. H. , Eldreth D. L. , Hyde C. , Matochik J. A. , London E. D. , Contoreggi C. *et al.* Risky decision making and the anterior cingulate cortex in abstinent drug abusers and nonusers. Brain Res Cogn Brain Res 2005; 23: 119–36. 15795139 10.1016/j.cogbrainres.2004.12.010

[85] Nestor L. J. , Ghahremani D. G. , Monterosso J. , London E. D. Prefrontal hypoactivation during cognitive control in early abstinent methamphetamine‐dependent subjects. Psychiatry Res 2011; 194: 287–95. 22047731 10.1016/j.pscychresns.2011.04.010 PMC3225642

[86] Kaufman J. N. , Ross T. J. , Stein E. A. , Garavan H. Cingulate hypoactivity in cocaine users during a GO–NOGO task as revealed by event‐related functional magnetic resonance imaging. J Neurosci 2003; 23: 7839–43. 12944513 10.1523/JNEUROSCI.23-21-07839.2003 PMC6740597

[87] Preller K. H. , Herdener M. , Schilbach L. , Stampfli P. , Hulka L. M. , Vonmoos M. *et al.* Functional changes of the reward system underlie blunted response to social gaze in cocaine users. Proc Natl Acad Sci USA 2014; 111: 2842–7. 24449854 10.1073/pnas.1317090111 PMC3932870

[88] Muraven M. Practicing self‐control lowers the risk of smoking lapse. Psychol Addict Behav 2010; 24: 446–52. 20853930 10.1037/a0018545 PMC2946169

[89] Schoenmakers T. M. , de Bruin M. , Lux I. F. , Goertz A. G. , Van Kerkhof D. H. , Wiers R. W. Clinical effectiveness of attentional bias modification training in abstinent alcoholic patients. Drug Alcohol Depend 2010; 109: 30–6. 20064698 10.1016/j.drugalcdep.2009.11.022

[90] Shoptaw S. , Heinzerling K. G. , Rotheram‐Fuller E. , Kao U. H. , Wang P. C. , Bholat M. A. *et al.* Bupropion hydrochloride versus placebo, in combination with cognitive behavioral therapy, for the treatment of cocaine abuse/dependence. J Addict Dis 2008; 27: 13–23. 10.1300/J069v27n01_02 18551884

[91] Goldstein R. Z. , Woicik P. A. , Maloney T. , Tomasi D. , Alia‐Klein N. , Shan J. *et al.* Oral methylphenidate normalizes cingulate activity in cocaine addiction during a salient cognitive task. Proc Natl Acad Sci USA 2010; 107: 16667–72. 20823246 10.1073/pnas.1011455107 PMC2944718

[92] Longo M. , Wickes W. , Smout M. , Harrison S. , Cahill S. , White J. M. Randomized controlled trial of dexamphetamine maintenance for the treatment of methamphetamine dependence. Addiction 2010; 105: 146–54. 19839966 10.1111/j.1360-0443.2009.02717.x

[93] Anton R. F. , Myrick H. , Wright T. M. , Latham P. K. , Baros A. M. , Waid L. R. *et al.* Gabapentin combined with naltrexone for the treatment of alcohol dependence. Am J Psychiatry 2011; 168: 709–17. 21454917 10.1176/appi.ajp.2011.10101436 PMC3204582

[94] Paulus M. P. , Tapert S. F. , Schuckit M. A. Neural activation patterns of methamphetamine‐dependent subjects during decision making predict relapse. Arch Gen Psychiatry 2005; 62: 761–8. 15997017 10.1001/archpsyc.62.7.761

[95] Stewart J. L. , Connolly C. G. , May A. C. , Tapert S. F. , Wittmann M. , Paulus M. P. Striatum and insula dysfunction during reinforcement learning differentiates abstinent and relapsed methamphetamine‐dependent individuals. Addiction 2014; 109: 460–71. 24329936 10.1111/add.12403 PMC3945155

[96] Gowin J. L. , Ball T. M. , Wittmann M. , Tapert S. F. , Paulus M. P. Individualized relapse prediction: personality measures and striatal and insular activity during reward‐processing robustly predict relapse. Drug Alcohol Depend 2015; 152: 93–101. 25977206 10.1016/j.drugalcdep.2015.04.018 PMC4458160

[97] Gowin J. L. , Harle K. M. , Stewart J. L. , Wittmann M. , Tapert S. F. , Paulus M. P. Attenuated insular processing during risk predicts relapse in early abstinent methamphetamine‐dependent individuals. Neuropsychopharmacology 2014; 39: 1379–87. 24317375 10.1038/npp.2013.333 PMC3988541

[98] Loughead J. , Wileyto E. P. , Ruparel K. , Falcone M. , Hopson R. , Gur R. *et al.* Working memory‐related neural activity predicts future smoking relapse. Neuropsychopharmacology 2015; 40: 1311–20. 25469682 10.1038/npp.2014.318 PMC4397393

[99] Cousijn J. , Wiers R. W. , Ridderinkhof K. R. , van den Brink W. , Veltman D. J. , Goudriaan A. E. Effect of baseline cannabis use and working‐memory network function on changes in cannabis use in heavy cannabis users: a prospective fMRI study. Hum Brain Mapp 2014; 35: 2470–82. 24038570 10.1002/hbm.22342 PMC6869744

[100] Janes A. C. , Pizzagalli D. A. , Richardt S. , de B. F. B. , Chuzi S. , Pachas G. *et al.* Brain reactivity to smoking cues prior to smoking cessation predicts ability to maintain tobacco abstinence. Biol Psychiatry 2010; 67: 722–9. 20172508 10.1016/j.biopsych.2009.12.034 PMC2954596

[101] Mann K. , Vollstadt‐Klein S. , Reinhard I. , Lemenager T. , Fauth‐Buhler M. , Hermann D. *et al.* Predicting naltrexone response in alcohol‐dependent patients: the contribution of functional magnetic resonance imaging. Alcohol Clin Exp Res 2014; 38: 2754–62. 25421512 10.1111/acer.12546

[102] Lenglet C. , Abosch A. , Yacoub E. , De Martino F. , Sapiro G. , Harel N. Comprehensive *in vivo* mapping of the human basal ganglia and thalamic connectome in individuals using 7 T MRI. PLOS ONE 2012; 7: e29153. 10.1371/journal.pone.0029153 PMC3250409 22235267

[103] Tofts P. S. , Collins D. J. Multicentre imaging measurements for oncology and in the brain. Br J Radiol 2011; 84: S213–26. 22433831 10.1259/bjr/74316620 PMC3473901

[104] Deoni S. C. , Williams S. C. , Jezzard P. , Suckling J. , Murphy D. G. , Jones D. K. Standardized structural magnetic resonance imaging in multicentre studies using quantitative T1 and T2 imaging at 1.5 T. Neuroimage 2008; 40: 662–71. 18221894 10.1016/j.neuroimage.2007.11.052

[105] Weiskopf N. , Suckling J. , Williams G. , Correia M. M. , Inkster B. , Tait R. *et al.* Quantitative multi‐parameter mapping of R1, PD(*), MT, and R2(*) at 3 T: a multi‐center validation. Front Neurosci 2013; 7: 95. 23772204 10.3389/fnins.2013.00095 PMC3677134

[106] Wolff S. D. , Balaban R. S. Magnetization transfer contrast (MTC) and tissue water proton relaxation *in vivo* . Magn Reson Med 1989; 10: 135–44. 2547135 10.1002/mrm.1910100113

[107] Reichenbach J. R. , Venkatesan R. , Schillinger D. J. , Kido D. K. , Haacke E. M. Small vessels in the human brain: MR venography with deoxyhemoglobin as an intrinsic contrast agent. Radiology 1997; 204: 272–7. 9205259 10.1148/radiology.204.1.9205259

[108] Mathew R. J. , Wilson W. H. Substance abuse and cerebral blood flow. Am J Psychiatry 1991; 148: 292–305. 1992832 10.1176/ajp.148.3.292

[109] Kaplan E. H. , Gottesman R. F. , Llinas R. H. , Marsh E. B. The association between specific substances of abuse and subcortical intracerebral hemorrhage versus ischemic lacunar infarction. Front Neurol 2014; 5: 174. 25309502 10.3389/fneur.2014.00174 PMC4159993

[110] Thomas B. P. , Welch E. B. , Niederhauser B. D. , Whetsell W. O., Jr. , Anderson A. W. , Gore J. C. *et al.* High‐resolution 7 T MRI of the human hippocampus *in vivo* . J Magn Reson Imaging 2008; 28: 1266–72. 18972336 10.1002/jmri.21576 PMC2669832

[111] Eapen M. , Zald D. H. , Gatenby J. C. , Ding Z. , Gore J. C. Using high‐resolution MR imaging at 7 T to evaluate the anatomy of the midbrain dopaminergic system. Am J Neuroradiol 2011; 32: 688–94. 21183619 10.3174/ajnr.A2355 PMC3076524

[112] Werner P. , Barthel H. , Drzezga A. , Sabri O. Current status and future role of brain PET/MRI in clinical and research settings. Eur J Nucl Med Mol Imaging 2015; 42: 512–26. 25573629 10.1007/s00259-014-2970-9

[113] Fox P. T. , Lancaster J. L. , Laird A. R. , Eickhoff S. B. Meta‐analysis in human neuroimaging: computational modeling of large‐scale databases. Annu Rev Neurosci 2014; 37: 409–34. 25032500 10.1146/annurev-neuro-062012-170320 PMC4782802

[114] Di Martino A. , Yan C. G. , Li Q. , Denio E. , Castellanos F. X. , Alaerts K. *et al.* The autism brain imaging data exchange: towards a large‐scale evaluation of the intrinsic brain architecture in autism. Mol Psychiatry 2014; 19: 659–67. 23774715 10.1038/mp.2013.78 PMC4162310

[115] Mueller S. G. , Weiner M. W. , Thal L. J. , Petersen R. C. , Jack C. R. , Jagust W. *et al.* Ways toward an early diagnosis in Alzheimer's disease: the Alzheimer's Disease Neuroimaging Initiative (ADNI). Alzheimers Dement 2005; 1: 55–66. 17476317 10.1016/j.jalz.2005.06.003 PMC1864941

